# Pitch-induced illusory percepts of time

**DOI:** 10.3758/s13414-024-02982-8

**Published:** 2024-12-10

**Authors:** Jesse K. Pazdera, Laurel J. Trainor

**Affiliations:** 1 Department of Psychology, Neuroscience and Behaviour, McMaster University, Hamilton, ON Canada; 2 McMaster Institute for Music and the Mind, McMaster University, Hamilton, ON Canada; 3 Rotman Research Institute, Baycrest Hospital, Toronto, ON Canada

**Keywords:** Illusion, Perceptual bias, Perceptual interaction, Pitch, Tempo, Time perception

## Abstract

Past research suggests that pitch height can influence the perceived tempo of speech and music, such that higher-pitched signals seem faster than lower-pitched ones. However, previous studies have analyzed perceived tempo across a relatively limited range of fundamental frequencies. To investigate whether this higher-equals-faster illusion generalizes across the wider range of human hearing, we conducted a series of five experiments. We asked participants to compare the tempo of repeating tones from six different octaves and with 15 different interonset intervals to a metronomic standard tempo. In Experiments 1–3, we observed an inverted U-shaped effect of pitch on perceived tempo, with the perceived tempo of piano tones peaking between A4 (440 Hz) and A5 (880 Hz) and decreasing at lower and higher frequencies. This bias was consistent across base tempos and was only slightly attenuated by synchronous tapping with the repeating tones. Experiment 4 tested synthetic complex tones to verify that this nonlinearity generalizes beyond the piano timbre and that it was not related to the presence of low-frequency mechanical noise present in our piano tones. Experiment 5 revealed that the decrease in perceived tempo at extremely high octaves can be abolished by exposing participants to only high-pitched tones. Together, our results suggest that perceived tempo depends more on the relative pitch within a context than on absolute pitch and that tempo biases may invert or taper off beyond a two-octave range. We relate this context-dependence to human vocal ranges and propose that illusory tempo effects are strongest within pitch ranges consistent with human vocalization.

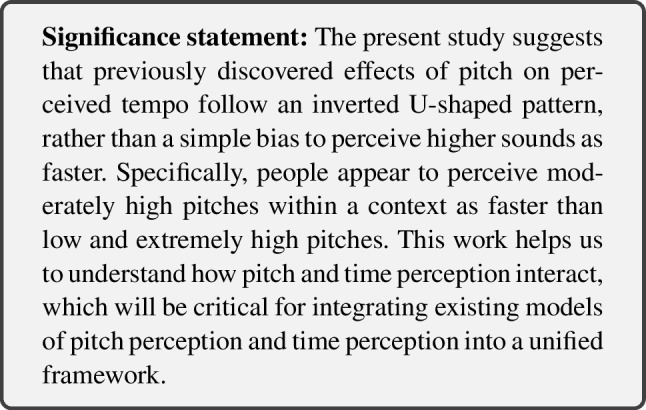



Accurate tempo tracking in auditory perception helps us to direct our attention to critical moments in speech and music (Jones, 1976; Jones & Boltz, 1989; Large & Jones, 1999), and can help us to interpret and communicate emotion (Scherer & Oshinsky, 1977; Scherer, 1986). However, past research suggests that the nontemporal features of an auditory stimulus can distort perceived tempo, (e.g., Boltz, 1998, 2011), through a phenomenon we will refer to as *illusory tempo*. In the present study, we investigated how the pitch of an auditory signal biases perceived tempo. In particular, we considered previous observations that people perceive higher-pitched signals to be faster than lower-pitched signals (Boltz, 2011; Collier & Hubbard, 1998), and we tested whether these effects generalize across a wider range of octaves and base tempos than previously evaluated.

## Illusory tempo effects

Using a paradigm in which participants listened to pairs of melodies and rated their relative tempos, Boltz (1998) showed that melodies with more contour changes or larger pitch intervals were perceived as slower than those with fewer contour changes or smaller pitch intervals. Boltz (2011) later demonstrated additional illusory tempo effects in which melodies played in a higher octave, brighter timbre, or which ascended in pitch or increased in loudness were perceived as faster than those played in a lower octave, duller timbre, or which descended in pitch or loudness. The perception of high-pitched or ascending sequences as speeding up, relative to low or descending sequences, has also been demonstrated in musical scales (Collier & Hubbard, 1998), pitch glides (Gordon & Ataucusi, 2021), and amplitude-modulated tones (Herrmann & Johnsrude, 2018). Consistent with this “higher-equals-faster” illusion, research on perceived interval duration has additionally shown that intervals flanked by at least one high-pitched tone tend to be underestimated, whereas those flanked by at least one low-pitched tone are overestimated (Lake et al., 2014; Pfeuty & Peretz, 2010). In contrast, higher-pitched sounds tend to be perceived as longer than lower-pitched sounds of the same duration (Brigner, 1988; Cohen et al., 1954; Gussenhoven & Zhou, 2013). Meanwhile, research on tempo preferences suggests that listeners prefer higher-pitched melodies to be played faster than lower-pitched melodies, with musical training strengthening this effect (Tamir-Ostrover & Eitan, 2015).

Implicit associations between higher pitch and faster tempo have been established in the literature, as well. A Stroop task by Walker and Smith (1984) found faster reaction times when high tones were paired with the words “fast” and “active” and low tones were paired with “slow” and “passive” than for the reverse pairings. Meanwhile, Eitan and colleagues have shown an association between higher pitch and faster imagined motion in adults (Eitan & Granot, 2006), although not reliably in children (Eitan & Tubul, 2010; Kohn & Eitan, 2009, 2016).

Beyond the domain of music, some evidence suggests that illusory tempo effects also apply to speech. Feldstein and Bond (1981) found that louder or higher-pitched speech was perceived as faster than quieter or lower-pitched speech, with pitch having the strongest effect when speech was quiet. Furthermore, Boltz (2017) found that participants were better able to identify changes in the pitch or tempo of a previously heard speech sample when both pitch and tempo were changed in the same direction, as compared to when the complementary feature was changed in the opposite direction or left unchanged, suggesting that the brain encodes pitch and tempo in an integrated manner.

Several sensorimotor studies suggest that illusory tempo also affects motor action. Ammirante et al. (2011) found in a synchronization-continuation tapping task that people tapped slower following tones that changed the pitch contour than following contour-preserving tones, consistent with the perceptual findings from Boltz (1998); however, they also observed faster tapping rates with larger pitch intervals, inverting the previously noted bias to rate melodies with larger intervals as slower. Synchronization tapping studies by Boasson and Granot (2012, 2019) have similarly shown decreases in tapping rates following contour changes, and have also demonstrated that both tapping rate and negative mean asynchrony increase when tapping to sequences that rise in pitch or intensity, which is consistent with the perceptual ratings from Boltz (2011).

Finally, evidence from Herrmann et al. (2013) suggests that illusory tempo effects may directly influence the dynamics of neural entrainment to a periodic signal. They demonstrated using MEG that the phase lag of stimulus-entrained oscillations in the right auditory cortex shift based on the pitch direction of a frequency-modulated tone. Furthermore, they observed increased intertrial phase coherence when an accelerating or decelerating modulation rate was paired with a congruent (increasing or decreasing) change in pitch, compared to an incongruent one. Together, these results suggest that changes in pitch can alter the relative phase of entrained oscillations.

**Fig. 1:**
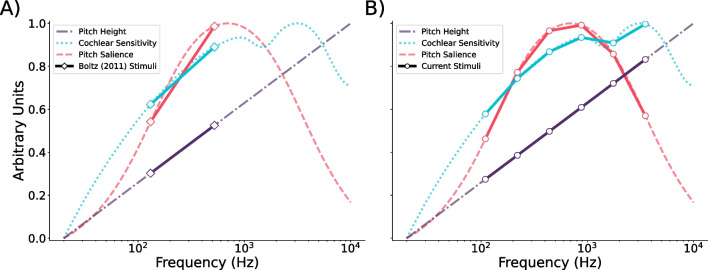
Factors varying across octaves. An illustration of the benefit of testing pitches spread across several octaves. *Background curves* illustrate normalized values of pitch height, cochlear sensitivity (based on the equal-loudness contour of ISO 226:2003; International Organization for Standardization, 2003), and pitch salience (based on Terhardt et al., 1982). *Points* denote A) the fundamental frequencies of the initial tones of melodies used by Boltz (2011), and B) those of the tones used in Experiments 1–4 of the present study. Pitch height, cochlear sensitivity, and pitch salience are all greater in the high-pitched melody condition of Boltz (2011) than in their low-pitched condition (**A**), and each could account for the increased perceived tempo of their high-pitched melodies. These factors can be disambiguated by sampling across several octaves (**B**)

## Origins of illusory tempo

Several potential explanations for illusory tempo effects have been proposed. Boltz (1998) has suggested an imputed velocity hypothesis, in which listeners apply expected timing onto stimuli based on learned associations between timing and acoustic features. For instance, melodic phrase boundaries typically coincide with temporal accents; therefore, the imputed velocity hypothesis suggests that listeners will perceive elongated timing at phrase boundaries regardless of whether a temporal accent was actually present (see also Henry and McAuley, 2009, for evidence of imputed velocity in the perceived timing of pitch changes). Likewise, correlations between pitch and tempo have been found in Western music, with higher-pitched voice lines tending to be faster than lower-pitched ones (Broze & Huron, 2013). It might therefore be the case that listeners impute faster timing onto melodies played in higher octaves because higher-pitched melodies are expected to be fast. Furthermore, studies of music visualization have found that adults (Eitan & Granot, 2006) associate rising pitch with spatial acceleration, which is consistent with rising lines being perceived as faster than falling lines (Boltz, 2011).

Alternatively, Ammirante et al. (2011) has proposed an ideomotor hypothesis, in which perceptual and motor representations of action interact. According to their hypothesis, the perceived tempo of an auditory object can be influenced by movements associated with that sound. For example, perceived slowing at contour changes may originate from the knowledge that bodies must slow down when changing direction. Likewise, faster tapping in response to larger pitch intervals may reflect the idea that one must move faster to travel longer distances in a fixed amount of time (Ammirante et al., 2011).

To explain the effects they observed on neural dynamics, Herrmann et al. (2013) suggested that the brain may recruit oscillators with different natural frequencies to track stimuli with different pitch directions. This hypothesis resembles an earlier proposal by Large (2000) that banks of rhythm-tracking oscillators could integrate pitch information by allowing higher-pitched sounds to preferentially stimulate naturally faster oscillators. In dynamical systems models of rhythm perception, neural oscillators entrain to the frequency at which they are stimulated while remaining attracted to their intrinsic natural frequency (Large & Snyder, 2009; Large et al., 2015). Therefore, if oscillators with different natural frequencies track stimuli with different pitches or pitch directions, the pitch may bias neural entrainment towards earlier or later phases, as well as faster or slower tempos (see Kim and Large, 2015 for an analysis of how oscillatory dynamics depend on the relation between the natural frequency of an oscillator and the tempo of its stimulus).

## The present study

Although there are many nontemporal factors known to influence perceived tempo, the present study focuses specifically on the effect of pitch height. In particular, we seek to address some of the limitations of the previous illusory tempo study conducted by Boltz (2011), in which people judged the relative tempo of differently pitched melodies. Although they concluded that higher-pitched melodies are perceived as faster than lower-pitched melodies, they compared only one lower register (melodies starting on C3 or 130.8 Hz) to one higher register (melodies starting on C5 or 523.3 Hz). This two-condition design leaves open the question of whether perceived tempo truly increases monotonically with pitch height, or whether it may vary non-monotonically across octaves.

In addition to our concerns over the generalizability of higher pitches being perceived as faster, it must be noted that confounding factors other than pitch height vary between octaves. Figure 1 illustrates pitch height alongside two such factors, cochlear sensitivity (related to the perception of loudness; Fletcher and Munson, 1933; International Organization for Standardization [ISO], 2003) and pitch salience (Huron & Parncutt, 1993; Terhardt et al., 1982). Like pitch height, both cochlear sensitivity and pitch salience are greater at C5 than at C3 (Fig. 1A). However, cochlear sensitivity exhibits substantial nonlinearities across octaves, and pitch salience follows an inverted U-shape that peaks near 700 Hz. Although we agree that pitch height is the most parsimonious explanation of earlier findings, we cannot rule out other potential factors based on existing data.

The present study seeks to address both limitations by testing perceived tempo at pitch heights spanning a five-octave range (Fig. 2B), from A2 (110 Hz) to A7 (3520 Hz). This procedure allows us to map out the shape of pitch-induced illusory tempo across the majority of the range within which humans perceive pitch most clearly. Mapping the effect across several octaves will confirm whether perceived tempo varies monotonically with pitch height, and will clarify whether pitch-induced illusory tempo is best explained by pitch height or a different factor that varies across octaves. We hypothesized that the relation between pitch and perceived tempo would indeed be monotonic, with higher pitches perceived as faster across a wide range of fundamental frequencies, thereby supporting the generalizability of the higher-equals-faster illusion.

## Experiments 1–3

Our first three experiments sought to determine the shape of the pitch-induced illusory tempo effect across a wide pitch spectrum and across a broad range of stimulus tempos. We did so using a relative tempo judgment paradigm in which participants heard a standard metronome sequence followed by an isochronous repeating tone, and then rated on a sliding scale how fast the repeating tone was relative to the standard. The repeating tone varied both in pitch and tempo across trials. We further investigated whether pitch-induced illusory tempo can be resisted through sensorimotor synchrony, as prior literature suggests movement can improve temporal judgments in some cases (Butler & Trainor, 2015; Manning & Schutz, 2013, 2016) but not others (London et al., 2019). Specifically, some participants were instructed to tap in time with the stimuli while others were instructed to minimize their movements while listening.

Procedurally, these three experiments were nearly identical, but each used a different stimulus tone design. Experiment 1 presented participants with 200-ms piano tones, but these tones varied naturalistically in their sustain, with higher tones tending to have sharper decays than lower tones. Experiment 2 reduced variability in the tones’ amplitude envelopes between octaves by presenting truncated, 50-ms piano tones. Experiment 3 returned to the longer, 200-ms tone design of Experiment 1 but guaranteed that tones sustained for the full 200 ms regardless of pitch. For conciseness, due to the similarity between these experiments, and in order to increase our power to detect interaction effects between pitch, stimulus tempo, and tapping behavior, we have pooled the data from these three experiments into a single analysis.

### Methods

#### Participants

Across Experiments 1–3, a total of 193 undergraduate students (58 male, 135 female) from McMaster University participated for course credit. The participant counts for each of the three experiments were 44 (25 female), 78 (51 female), and 71 (59 female), respectively. Ages ranged from 16–31 years, with mean ages of 18.9 ($SD=1.3$), 19.8 ($SD=2.1$), and 18.2 ($SD=1.3$) years across the three experiments, respectively. Within each experiment, participants were randomly assigned to either the tapping or non-tapping condition, with a total of 97 (71 female) assigned to the tapping condition and 96 (64 female) assigned to the non-tapping condition. We conducted the experiments sequentially between April and September 2020, and individuals were not permitted to participate in more than one of the three experiments. All participants completed the experiments online due to the COVID-19 pandemic.

An additional 16 participants completed the study but were excluded using the following criteria. Ten participants were excluded for treating the slider as a discrete response scale. We classified participants as discrete-responders if they responded with a rating of 0, 50, or 100 on at least 75 out of 90 trials. We excluded an additional six participants for having a low or negative correlation ($r<.5$) between their responses and the true relative tempo of the stimulus (see Response scoring for the calculation of ground-truth tempo ratings).

#### Data availability

We have made all data, code, and stimuli from all five experiments publicly available on the Open Science Framework at https://osf.io/85cyx/, as well as on GitHub at https://github.com/jpazdera/IllusoryTempo.

#### Materials

In Experiments 1 and 2, we generated the seven unique tones used in the study (A2, A3, A4, A5, A6, A7, and D$\sharp$5), as well as the sound of the metronome, using the music composition software MuseScore. The metronome tick was a simulated wood block sound lasting approximately 50 ms, and the tones were piano notes that varied in duration prior to editing. We used Python (version 3.7) to convert all eight sounds from stereo to mono, truncate all piano tones to either a 200-ms (Experiment 1) or 50-ms duration (Experiment 2), and apply a 10-ms linear fade-out to each tone. In Experiment 3, we used the same metronome as Experiments 1 and 2, but generated a new set of piano tones using Sonic Pi (version 3.2) to ensure that all tones sustained longer than 200 ms. We then used Python to convert the tones from stereo to mono, truncate them to a 200-ms duration, and apply a 25-ms linear fade-out.

We additionally equalized the perceived loudness of the tones in each experiment to limit the potential effects of loudness differences on perceived tempo (Boltz, 2011). In Experiment 1, we equalized the baseline loudness of all tones to -16 LKFS using the *pyloudnorm* package for Python (Steinmetz, 2019), which implements ITU-R BS.1770-4 (International Telecommunications Union [ITU], 2017). In Experiments 2 and 3, we instead used the |acousticLoudness| function in MATLAB (version R2020a) to equalize the perceived loudness of all tones according to ISO 532-1:2017 (ISO, 2017). For each experiment, we then used the audio editing software Audacity to generate louder ($+3$ dB) and softer ($-3$ dB) variants of each tone for a total of 21 piano tone stimuli.

To ensure precise inter-onset timing, we pre-generated all sound sequences using Python and played them back as WAV files during the experiment.

#### Apparatus

We used the JavaScript library jsPsych (de Leeuw, 2015) to implement our stimulus presentation and response collection, and conducted the experiment online through Pavlovia (https://pavlovia.org). We performed all analyses using Python (version 3.10) and R (version 4.3).

#### Design

The study followed a mixed 2 (Tap Instruction) $\times$ 6 (Pitch Height) $\times$ 15 (Stimulus Tempo) design. Participants assigned to the tapping condition were instructed to tap the J or F key with their dominant hand in synchrony with both the metronome and repeating tone on each trial. Participants in the non-tapping condition were instead instructed to listen to both sequences while keeping their movements to a minimum. In Experiments 2 and 3, reminder text was also displayed (“Remember to [tap along/avoid moving] while listening.”) while the metronome and repeating tone played on each trial. Each trial used one of six pitch heights for its repeating tone: A2 (110 Hz), A3 (220 Hz), A4 (440 Hz), A5 (880 Hz), A6 (1760 Hz), or A7 (3520 Hz). The interonset interval (IOI) of the repeating tone was set to one of 15 log-spaced values on each trial: 1000, 918, 843, 774, 710, 652, 599, 550, 504, 463, 425, 390, 358, 329, or 302 ms. These intervals correspond to the following tempos in beats per minute (BPM): 60.0, 65.4, 71.2, 77.5, 84.5, 92.0, 100.2, 109.1, 119.0, 129.6, 141.2, 153.8, 167.6, 182.4, and 199.7. We chose to center the timing conditions around 550 ms (109.1 BPM), as this tempo falls approximately where rhythm perception exhibits the greatest sensitivity and least bias (e.g., Drake & Botte, 1993; Friberg & Sundberg, 1995; Vos et al., 1997). We then selected an overall range of between 60 and 200 BPM to provide good coverage of the tempos most commonly observed in music, while keeping the rate solidly within the limits of human performance (Repp, 2006). For certain analyses, as well as during trial randomization, these 15 stimulus tempos were binned into five “tempo ranges” based on their quintile, such that 1000, 918, and 843 ms comprised the slowest tempo range, 774, 710, and 652 ms comprised the second slowest range, and so forth. On practice trials, the pitch of the repeating tone was D$\sharp$5 (622.25 Hz, the midpoint of A2 and A7) and the interonset interval on each trial was one of 741, 550, and 407 ms.

Given the variability in online participants’ audio equipment – which may have affected the relative loudness of different pitches depending on their specific hardware – we additionally manipulated tone loudness as a control variable, in order to test whether small differences in intensity may affect perceived tempo. The repeating tone on each trial was either played at its baseline loudness, 3 dB louder than baseline, or 3 dB quieter than baseline.

#### Procedure

Before receiving instructions for the main task, participants completed a headphone test based on that of Woods et al. (2017). The test consisted of six trials in which participants listened to three 200-Hz pure tones and rated which was quietest. Two of the tones were played with equal loudness, while the target was played at 6 dB below that level. However, one of the equal-loudness tones was phase-reversed between stereo channels, making it sound quieter than the other when played over speakers. Consequently, participants wearing headphones reliably identify the target tone as quietest, while those listening through speakers do not (Woods et al., 2017).

Following the headphone test, we measured each participant’s spontaneous motor tempo (McAuley et al., 2006) by asking them to rest their palms on the surface in front of them and tap with the index finger of their dominant hand on the J or F key at the rate that felt most natural and comfortable to them. After 20 (Experiment 1) or 15 (Experiments 2 and 3) seconds of tapping, participants were stopped and given the main task instructions.

Each trial featured a relative tempo judgment task in which participants listened to five ticks of a metronome followed by five repetitions of a piano tone. The metronome was identical on all trials and always played with an interonset interval of 550 ms, whereas the tone varied across trials in pitch, interonset interval, and loudness. An interval of 1800 ms of silence separated the metronome from the repeating tone on all trials. Following the repeating tone, participants were given a continuous slider and asked “How fast was the repeating tone relative to the metronome?” The slider’s scale ranged from “Half as Fast” (a score of 0) on the left to “Twice as Fast” (a score of 100) on the right, with “Equal Rates” (a score of 50) labeled in the center. Participants could only see the descriptive labels, and were not able to view the numeric values assigned to their responses. Participants were not limited in how long they could take to respond, and the next trial began 1500 ms after they submitted a response.

Each participant completed three practice trials, as well as one trial under each of the 90 unique pitch $\times$ tempo pairings. Among the three presentations of each pitch within each of the five “tempo ranges,” one trial was played at each of the three loudness levels. Trials were arranged into three blocks of 30 and were randomly ordered, with the constraint that each pitch was paired with one tempo from each of the five tempo ranges exactly once per block. Additionally, consecutive trials within a block always differed in both pitch and tempo range. Participants were allowed self-paced breaks between blocks.

#### Response scoring

**Fig. 2:**
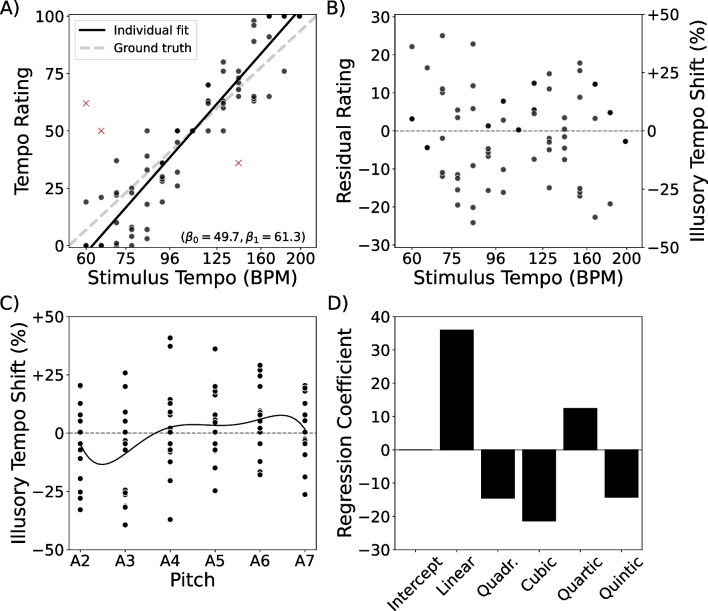
Example data processing. Data processing steps for one example participant. **A)** A line is fit to the participant’s raw tempo ratings (*black dots*) as a function of the log of the stimulus tempo (Eq. 2). After an initial fit, outlier ratings (*red x’s*) are identified and excluded, and the line is refit. Ground-truth relative tempo, based on the labeling of the response scale, is shown for comparison (*dashed line*; Eq. 1). **B)** The effect of stimulus tempo is regressed out by subtracting the expected rating for each trial from the participant’s response, giving a set of residual tempo ratings (Eq. 4). Residual tempo ratings are then converted to a percent illusory tempo shift by dividing the residuals by the slope of the regression line. **C)** A polynomial regression is fit to predict the participant’s illusory tempo shift as a function of pitch height. **D)** The subject-level regression coefficients are extracted, and their distributions across participants are later analyzed to identify the shape of the illusory tempo curve. Shown here are the regression coefficients from one participant. Note that the polynomial’s intercept equals zero due to having already regressed out the average rating using Eq. 4

##### Deriving perceived tempo from raw ratings

To facilitate the interpretation of the 0–100 ratings on the response scale, we first defined ground-truth ratings for all stimulus tempos. These ground-truth ratings were based on the assumption that – given the labeling of the response scale – a rating of 50 should correspond to a stimulus tempo equal to that of the metronome (i.e., 109.1 BPM or a 550 ms IOI), and a rating increase of 50 points should correspond to a doubling of the tempo (i.e., a halving of the IOI). This mapping assumes that tempo is perceived on a log-linear scale, which can be described in our study by the linear equation:

1 $$\begin{aligned} r_{true} = 50 + 50\log _2\Big (\frac{t}{t_{ref}}\Big ) \end{aligned}$$

where $r_{true}$ is the ground-truth rating for a given relative tempo, $t/t_{ref}$, where *t* is the stimulus tempo in BPM and $t_{ref}$ is the tempo of the metronome in BPM.^1^

However, we did not expect all participants to map relative tempo onto the response scale in a manner identical to the ground truth. For example, some individuals may use the full sliding scale liberally while others may give conservative judgments that deviate less from the midpoint of the scale. We therefore sought to account for individual differences in response scale usage when scoring our response data, before considering the effect of pitch on these ratings. To do so, we fit separate linear models to each participant’s responses, predicting their tempo ratings as a function of the log of the relative tempo of the stimulus:

2 $$\begin{aligned} \hat{r}_i = \beta _0 + \beta _1\log _2\Big (\frac{t_i}{t_{ref}}\Big ) \end{aligned}$$

where $t_{ref}$ is the tempo of the metronome, $t_i$ is the stimulus tempo on a given trial *i*, and $\hat{r}_i$ is the predicted rating the participant would assign to that stimulus tempo. The intercept, $\beta _0$, is their expected rating when the stimulus has the same tempo as the metronome, and the slope, $\beta _1$, describes how many points their rating is expected to change each time the stimulus tempo doubles.

After an initial fit of each participant’s linear model, we marked trials with Cook’s distance greater than 4/90 (where 90 is the trial count) as outliers, then refit the model with outliers excluded. The outlier-excluded slopes and intercepts were used for the remainder of our analyses, and the outlier trials (932 total, 5.4% of trials) were excluded from all further analyses. Figure 2A shows the tempo ratings from one example participant and illustrates the linear model mapping stimulus tempos to their associated ratings on the response scale. Also shown is the ground truth line from Eq. 1, for comparison.

Note that Eq. 2 can be inverted to estimate the perceived tempo, $\hat{t}_i$, associated with any given rating, $r_i$:

3 $$\begin{aligned} \hat{t}_i = t_{ref} * 2^{(r_i-\beta _0)/\beta _1} \end{aligned}$$

where $t_{ref}$ is the tempo of the metronome in BPM.

##### Quantifying illusory tempo shift

To address the question of how pitch influences perceived tempo, we analyzed whether participants’ responses to tones of each pitch differed systematically from the response that would be expected based solely on the tempo of those tones. We did so by regressing out the expected tempo ratings (Eq. 2) from the participants’ response values. We define the residual tempo rating, $\rho _i$, for trial *i* as the difference between the participant’s response on that trial, $r_i$, and the predicted rating, $\hat{r}_i$, for stimuli of that trial’s tempo:

4 $$\begin{aligned} \rho _i = r_i - \hat{r}_i \end{aligned}$$

Thus, a positive residual tempo rating indicates that the participant rated the repeating tone on that trial as faster than they would typically rate a stimulus of that tempo, whereas a negative residual tempo rating indicates that the participant rated the repeating tone as slower than would be expected. Figure 2B illustrates residual tempo ratings for one participant’s trials after regressing out the stimulus tempo. Note that the average residual rating within a participant will always equal zero, as we have regressed out the participant’s average rating.

To translate each participant’s residual tempo ratings into percent changes in perceived tempo, we divided the residuals by that person’s regression slope, $\beta _1$, from Eq. 2. As $\beta _1$ describes the number of rating points associated with a doubling in perceived tempo, our illusory tempo shifts should be understood as a percent of a *doubling* in BPM, not a percent change in raw BPM.^2^ This scaling follows the overarching assumption in our study that tempo is perceived on a log-linear scale. We thus define the illusory tempo shift, $\tau _i$, on a given trial, *i*, as:

5 $$\begin{aligned} \tau _i = 100\% * \frac{\rho _i}{\beta _1} \end{aligned}$$

The two *y*-axes of Fig. 2B illustrate the mapping of residual ratings to illusory tempo shift, based on the example participant’s regression slope of $\beta _1=61.3$.

Note that the above formulation of illusory tempo shift is equivalent to the $\log _2$-percent change in perceived tempo (see Törnqvist et al., 1985 for a discussion of log-percents):

6 $$\begin{aligned} \tau _i = 100\% * \log _2\Big (\frac{\hat{t}_i}{t_i}\Big ) \end{aligned}$$

where $t_i$ is the stimulus tempo on trial *i*, and $\hat{t}_i$ is the perceived tempo associated with the participant’s rating on that trial (see Eq. 3).

#### Data analysis

##### Comparing subjective ratings to the ground truth

As a preliminary analysis of the overall performance of participants in our task, we compared the slopes and intercepts of the subject-level linear models of tempo ratings as a function of stimulus tempo (Eq. 2) against the ground-truth model of relative tempo (Eq. 1). We did so using a one-sample Hotelling’s $T^2$ test (a multivariate *t*-test) with the intercepts and slopes both as outcome variables. This test assessed whether the average subject-level model differed from the ground-truth in terms of intercept ($\beta _0=50$) and/or slope ($\beta _1=50$). We then conducted post hoc univariate *t*-testing to determine which of these two model parameters significantly differed from the ground truth and used the Holm–Bonferroni technique to correct for multiple comparisons.

##### Assessing pitch-induced illusory tempo

To investigate how perceived tempo varied across octaves, we used polynomial regression as a method of quantifying the pattern of illusory tempo shift across our six levels of pitch height. We used orthogonal polynomials in all of these models to eliminate multicollinearity between regression coefficients. In all cases where we analyzed a polynomial regression model, we first performed multivariate statistics (Hotelling’s $T^2$ or MANOVA) with all model coefficients of interest as outcome variables. We did so as an omnibus test of whether the average of all subject-level models differed as a whole from a null model (in one-sample tests), or whether the average model differed between conditions (in two-sample tests). Then, in any case where a multivariate analysis produced a significant result, we conducted post hoc univariate *t*-testing to determine which specific model coefficients accounted for the significant effect. We corrected for multiple comparisons in each post hoc analysis using the Holm–Bonferroni technique.

##### Main effect of pitch

To evaluate the main effect of pitch height on perceived tempo, we analyzed how illusory tempo shift (Eq. 5) differed across octaves. To quantify the within-subject shape of the illusory tempo effect, we fit separate fifth-order polynomial regression models to each person’s data, predicting illusory tempo shift as a function of pitch height (coded as integers from 2 to 7, prior to making the polynomial factors orthogonal). Figure 2C illustrates the regression model for one example participant, and Fig. 2D shows the coefficients of this model. As every participant’s average residual tempo rating equaled zero (see Response scoring), the intercept of the illusory tempo polynomial also always equaled zero. Therefore, we analyzed only the distribution of slopes across participants. Specifically, we conducted a one-sample Hotelling’s $T^2$ test with the linear, quadratic, cubic, quartic, and quintic slopes of the polynomial regression models as the outcome variables. This test indicated whether the average model of pitch-induced illusory tempo shift differed from a null model in which all slopes were zero.

##### Effect of pitch across stimulus tempos

After quantifying the shape of the illusory tempo curve across octaves, we tested whether the effect of pitch height differed between the five stimulus tempo ranges. To do so, we fit five separate second-order polynomial regression models within each participant – one to their illusory tempo shift data from each tempo range. These models were of an order equal to the highest significant model order determined during our analysis of the main effect of pitch. This procedure gave linear and quadratic slopes describing the shape of the illusory tempo effect within each of the five stimulus tempo ranges. We then conducted a repeated-measures MANOVA with the five levels of tempo range as the independent variable and the linear and quadratic slopes as dependent variables. Multicollinearity was avoided through the use of orthogonal polynomials, as noted above. Multivariate normality was inspected graphically, which suggested higher than normal kurtosis but a symmetrical distribution, to which MANOVA should be robust. A Box’s *M* test indicated significant heterogeneity of covariances; however, our balanced design should allow robustness to such a violation, as well.

##### Effect of tapping on illusory tempo

To determine whether synchronous tapping attenuated the illusory tempo effect, we fit a second-order polynomial regression model to each participant’s illusory tempo shifts as a function of pitch height, while excluding trials on which participants who were instructed to tap failed to do so (27.6% of trials). We then conducted an independent-samples Hotelling’s $T^2$ test to determine whether the linear and quadratic slopes differed between participants in the two tapping conditions.

##### Effect of loudness on perceived tempo

Finally, we tested whether our control variable, loudness, affected the perceived tempo of the repeating tones. We did so using a repeated-measures ANOVA to identify any differences in average illusory tempo shift between tones played 3 dB above, below, or at baseline. A significant deviation from sphericity was detected via Mauchly’s test, and Huynh–Feldt correction was applied accordingly ($\varepsilon =.957$).

### Results

#### Subjective ratings versus ground truth

**Fig. 3:**
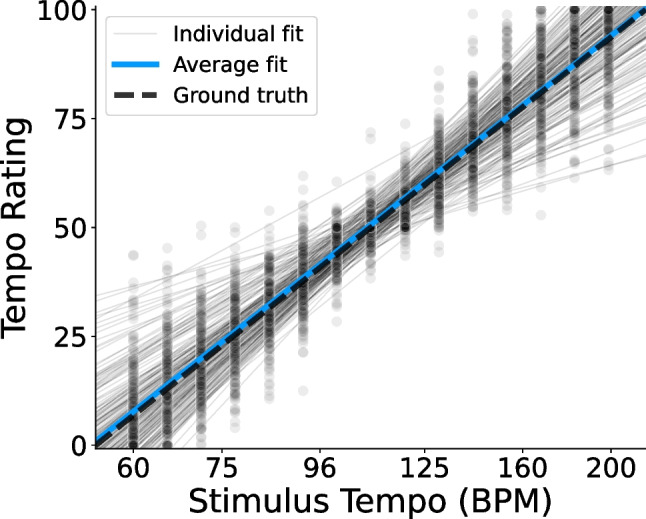
Raw tempo ratings in Experiments 1–3. *Points* show participants’ average ratings for each stimulus tempo in Experiments 1–3. Ground-truth ratings (Eq. 1) are indicated by the *dashed line*. Linear regression lines for individual participants (Eq. 2) appear as *thin gray lines*, and the average of all regression lines appears in *blue*

**Fig. 4:**
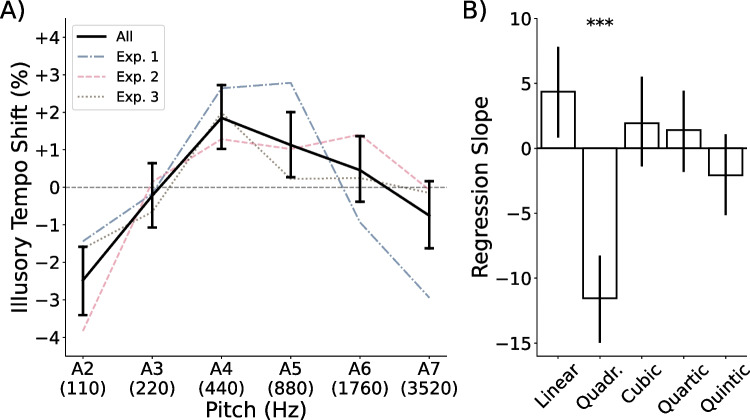
Illusory tempo by pitch height in Experiments 1–3. **A)** Average illusory tempo shifts across participants for repeating tones of each pitch height in Experiments 1–3. Perceived tempo followed an inverted U-shaped pattern, with extremely high and low tones rated as slower than middle tones. **B)** Average slope coefficients across subject-level polynomial regression models, predicting illusory tempo shift as a function of pitch height. *Error bars* in both panels indicate 95% confidence intervals

Figure 3 illustrates subject-level tempo ratings as a function of the repeating tone’s tempo, in comparison to the theoretical ground-truth ratings (dashed line; Eq. 1). Also shown is each participant’s individual regression line (thin gray lines; Eq. 2), used in deriving their residual tempo ratings and illusory tempo shift scores. The average of all individual fits is shown, as well (blue line). The average fit ($\beta _0=50.59$, $\beta _1=49.70$) differed significantly from the ground truth relative tempo, $F(2, 191)=3.50$, $p=.032$, $\eta _p^2=.035$, and post hoc testing revealed that the average intercept was significantly greater than the ground truth of 50, $t(192)=2.62$, $p=.009$, $d=0.189$, $CI=[50.15, 51.04]$. The average slope ($M=49.70$), however, did not differ significantly from the ground truth, $t(192)=-0.40, p=.693, d=0.029$, $CI=[48.20, 51.20]$. Though significant, the small difference between participants’ average behavior and the true relative tempo suggests that they were generally quite accurate at the task, but tended to rate the repeating tone as slightly faster than it actually was.

**Fig. 5:**
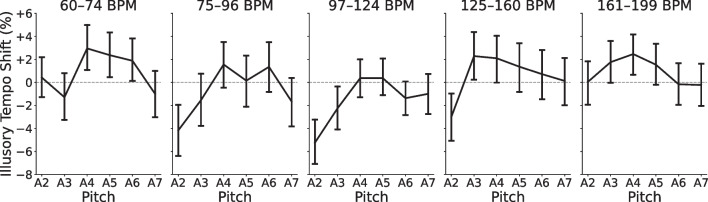
Illusory tempo effects by tempo range in Experiments 1–3. Average illusory tempo shifts across participants for repeating tones of each pitch height in Experiments 1–3, conditional on the true stimulus tempo. *Error bars* indicate within-subject 95% confidence intervals. Regardless of tempo range, the illusory effect of pitch consistently followed an inverted U-shaped pattern

**Fig. 6:**
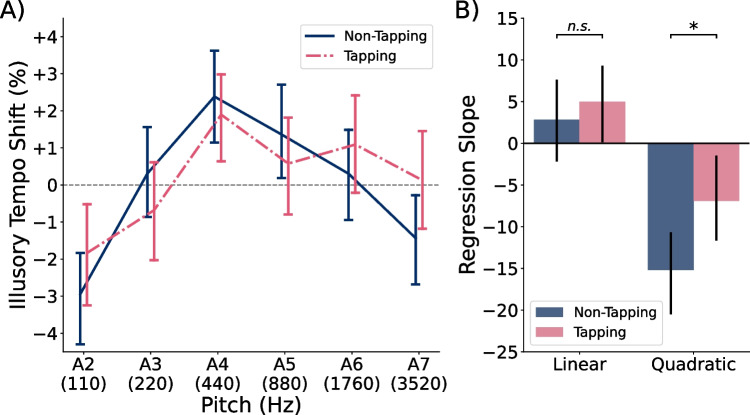
Sensorimotor synchrony and the illusory tempo effect in Experiments 1–3. **A)** Illusory tempo shifts for repeating tones of each pitch height, conditional on whether the participant was instructed to tap. Averages across participants instructed not to tap are shown in *blue*, while averages across participants instructed to tap are shown in *pink*. **B)** Average slope coefficients across subject-level polynomial regression models, predicting illusory tempo shift as a function of pitch height. *Error bars *in both panels indicate 95% confidence intervals. Synchronous tapping slightly attenuated the negative quadratic effect of pitch on perceived tempo

#### Pitch-induced illusory tempo

Our primary research question was how pitch height would affect participants’ tempo ratings across a wider frequency range than previously studied. Figure 4A illustrates the percent illusory tempo shift observed for repeating tones at each pitch height. Illusory tempo followed an inverted U-shaped pattern, in which the lowest (A2; 110 Hz) and highest (A7; 3520 Hz) tones were rated as slowest, whereas the middlemost tones (A4 and A5; 440 and 880 Hz) were rated as fastest. Figure 4B shows the average slope coefficients of the polynomial regression models fit to each participant’s illusory tempo data. A multivariate analysis of these coefficients revealed that pitch exerted a large and significant overall effect on perceived tempo, $F(5, 188)=10.51$, $p<.001$, $\eta _p^2=.218$. Post hoc testing indicated a marginal positive linear effect, $t(192)=2.40$, $p=.017$, $d=0.173$, and a significant negative quadratic effect of pitch height, $t(192)=-6.87$, $p<.001$, $d=0.494$. The cubic, $t(192)=1.10$, $p=.275$, $d=0.079$, quartic, $t(192)=0.49$, $p=.622$, $d=0.036$, and quintic effects of pitch height, $t(192)=-1.45$, $p=.149$, $d=0.104$, were all nonsignificant. A combined positive linear and negative quadratic effect is consistent with the slightly asymmetrical inverted-U shaped curve seen in Fig. 4A.

Next, we considered whether the effect of pitch might differ across stimulus tempo ranges. Figure 5 shows illusory tempo shift for each pitch, conditional on the tempo range of the repeating tone. The linear and quadratic slopes of the illusory tempo curve did not significantly differ between tempo ranges, Wilk’s $\Lambda =0.983$, $F(8, 1534)=1.65$, $p=.107$, $\eta _p^2=.009$, indicating a lack of interaction between pitch and stimulus tempo. Pitch influenced the perceived tempo of the repeating tone similarly, regardless of the tempo at which it was played.

Finally, we considered whether synchronous movement may attenuate the tempo-biasing effect of pitch. Figure 6A illustrates the effect of pitch among participants instructed to tap, and among those instructed to minimize their movements. The polynomial regression slopes for each condition are shown in Fig. 6B. We observed a small difference between the illusory tempo curve of participants instructed to tap and those instructed not to, $F(2, 181)=3.28$, $p=.040$, $\eta _p^2=.035$. The linear effect of pitch was not found to differ significantly between conditions, $t(182)=-0.64, p=.526, d=0.089$, but the quadratic effect of pitch was slightly weaker among participants in the tapping condition, $t(182)=-2.29, p=.023, d=0.330$.

#### Loudness control

Although we normalized all of our tones to be equally loud, our study was conducted online, where certain pitches may have sounded louder than others depending on participants’ hardware and individual hearing loss. To assess whether subtle loudness differences between pitches may have affected participants’ tempo ratings, we varied loudness across trials within a 6 dB range (see Methods). Loudness did not significantly affect tempo ratings, $F(1.91, 367.66)=0.79$, $p=.449$, $\eta _p^2=.004$. Average illusory tempo shifts for tones played at 3 dB below baseline, at baseline, and 3 dB above baseline were $+0.34\%$ ($SD=3.88$), $-0.17\%$ ($SD=3.29$), and $-0.17\%$ ($SD=4.04$), respectively. These small, nonsignificant differences in perceived tempo across a 6 dB range suggest that variability in the loudness of tones from different octaves is unlikely to have produced the effects of pitch that we observed.

### Discussion

Experiments 1–3 investigated the effect of pitch height on perceived tempo across tones ranging from A2 (110 Hz) to A7 (3520 Hz). They revealed an inverted U-shaped effect, such that the perceived tempo increased with pitch from A2 to A4 (440 Hz), then decreased with pitch between A4 and A7. This pattern was slightly asymmetrical, with a steeper upward than downward slope, characterized by a significant negative quadratic effect and a marginal positive linear effect. These results help to refine previous suggestions that perceived tempo increases monotonically with pitch (Boltz, 2011; Collier & Hubbard, 1998), and highlight the value of testing effects across several levels of continuous independent factors. Our results are consistent with the findings of Boltz (2011) within the range of fundamental frequencies tested in their study (see Fig. 1). Specifically, they compared the perceived tempo of melodies starting on C3 or C5 (130.8 Hz or 523.3 Hz; three semitones above A2 and A4, respectively) and found that participants perceived the higher-pitched melodies as faster. This design places their high-pitched melodies close to the point of fastest perceived tempo in our illusory tempo curve and their low-pitched melodies at a point where tempo was perceived to be slower in our illusory tempo curve (see Fig. 4). However, the results of our Experiments 1–3 suggest a non-monotonic relation between pitch and perceived tempo when the full frequency range we tested is considered.

We also tested whether the effect of pitch varied across stimulus tempos, as well as whether sensorimotor synchronization would attenuate pitch-induced biases. Our results suggest that the effect of pitch on perceived tempo is similar regardless of stimulus tempo. Across five tempo ranges with interonset intervals between 1000 ms (60.0 BPM) and 302 ms (199.7 BPM), we consistently found a similar inverted U-shaped effect (see Fig. 5). Regarding the effects of sensorimotor synchronization, we observed a small, but significant reduction in the quadratic effect of pitch on perceived tempo among participants who were instructed to tap in synchrony with the stimuli (see Fig. 6). In conjunction with previous research demonstrating illusory tempo effects on tapping rates during both synchronization (Boasson & Granot, 2012, 2019) and continuation tapping paradigms (Ammirante et al., 2011), we believe it is unlikely that pitch-induced timing biases can be entirely resisted through motor engagement, though weak attenuation may be possible.

As the observed inverted U-shaped effect of pitch was unexpected, we conducted two follow-up experiments in which we evaluated whether specific confounding variables may have driven the negative quadratic effect. One explanation is that we may be observing an effect of pitch salience, instead of (or in addition to) pitch height. Indeed, Fig. 4 somewhat resembles the pitch salience curve defined by Terhardt et al. (1982). Their model of pitch salience peaks at 700 Hz – approximately four semitones below A5 (880 Hz) – and declines symmetrically above and below this point (see Fig. 1). Furthermore, the effect of pitch height Experiments 1–3 may have been confounded with an effect of pitch salience because the piano tones we used contained some audible low-frequency content, deriving from the mechanics of the piano. This low-frequency content was audible as a subtle knocking sound that was particularly noticeable in our A6 and A7 tones – possibly due to the pitch of these tones being less salient relative to the knocking (Terhardt et al., 1982). If participants attended on some trials to the low-pitched knocking sound rather than to the highest-pitched piano tones, we might expect them to rate the highest tones as slower than they otherwise would. We therefore conducted Experiment 4 specifically to address whether the inverted U-shaped effect persists when participants listen to controlled, synthetic tones that lack low-frequency content.

An alternative possibility is that a midpoint effect influenced participants’ tempo ratings, such that they perceived tones in the middle of the pitch range to be the fastest. The fact that tempo ratings peaked between the middlemost pitches in our experiments (A4 and A5) supports this explanation. We conducted Experiment 5 to address the possibility of a midpoint effect by presenting each participant with six tones from only one of the two halves of our pitch range.

## Experiment 4

Experiment 4 addressed the possibility that the unexpected negative quadratic effect of pitch may be specific to the piano tones we tested in Experiments 1–3. To investigate whether the low-frequency content in our piano tones led to the decrease in perceived tempo at our highest octaves, we presented participants with the piano tones from Experiment 3 on some trials and controlled, synthetic complex tones on others. If the quadratic component depends on low-frequency content becoming more salient (Terhardt et al., 1982) relative to the tone at high octaves, the downturn in perceived tempo above 700 Hz should be eliminated for synthetic tones.

### Methods

#### Participants

Seventy-seven undergraduate students (27 male, 50 female) from McMaster University completed the experiment for course credit. Ages ranged from 18–24 years ($M=18.6$, $SD=1.2$). Participants were randomly assigned to hear piano tones on the first block ($N=36$; 21 female) or synthetic tones on the first block ($N=41$; 29 female). All participants completed the task online between February and April 2021 due to the COVID-19 pandemic. An additional three participants completed the experiment but were excluded due to low correlation ($r<.5$) between their responses and the true relative tempo (see Response Scoring).

#### Materials

The metronome was identical to that used in Experiments 1–3 and the piano tones were identical to those used in Experiment 3. The synthetic tones used in Experiment 4 were complex tones created in Python (version 3.8) by summing four sine waves with random phase, including the fundamental frequency and the first three overtones with relative amplitudes defined by a slope of -6 dB/octave. The tones were 200 ms in duration and consisted of a 5-ms linear rise, a 170-ms sustain, and a 25-ms linear decay, approximately matching the amplitude envelopes of the piano tones. We used Audacity’s loudness normalization function, which is based on recommendation ITU-R BS.1770-4 (ITU, 2017), to balance all tones to $-17$, $-14$, and $-11$ LUFS for the soft, normal, and loud versions of each tone, respectively.^3^ All sound sequences were again pre-generated using Python, and were played back as WAV files during the experiment.

#### Apparatus

We conducted the experiment online using the same apparatus as Experiments 1–3.

#### Design

Experiment 4 followed a 2 (Timbre) $\times$ 6 (Pitch Height) $\times$ 15 (Stimulus Tempo) fully within-subjects design. The repeating tone on each trial was either a synthetic complex tone or one of the piano tones used in Experiment 3. The pitch heights and stimulus tempos of the tones were identical to those used in Experiments 1–3, and tempos were again binned into the same five tempo ranges. Tone loudness was again included as a control variable, with the repeating tone either being played at its baseline loudness, at 3 dB above its baseline, or at 3 dB below its baseline.

#### Procedure

Participants began by completing the same headphone test used in Experiments 1–3 (Woods et al., 2017). They were then given the main task instructions. Each trial featured the same relative tempo judgment task from Experiments 1–3, with the exception that the tapping manipulation was removed. Each participant completed three practice trials, as well as one repetition of each of the 180 unique combinations of timbre, pitch height, and stimulus tempo. Among the three presentations of each timbre $\times$ pitch height $\times$ tempo range pairing, one trial was played at each of the three loudness levels. Trials were arranged into six blocks of 30. All trials within a block used the same timbre, and the timbre alternated between blocks in an ABABAB pattern. We randomized which timbre was presented in the first block between participants, and we randomized pitch heights and stimulus tempos using the same constraints as were used in Experiments 1–3. The practice trials matched the timbre used in the first block, and their pitch heights and tempos were identical to those used in the practice trials for Experiments 1–3.

#### Response scoring

Response scoring was identical to Experiments 1–3, with the exception that we reduced the Cook’s distance threshold for excluding trials as outliers from 4/90 to 4/180 due to the trial count doubling in Experiment 4. A total of 709 trials (5.12%) were excluded as outliers via this metric.

#### Data analysis

We applied the same general process from Experiments 1–3, in which we first analyzed all coefficients of our regression model together via a multivariate test, and then used univariate post hoc testing as necessary with Holm–Bonferroni correction. We tested the subject-level linear models of tempo ratings (Eq. 2) against ground-truth relative tempo (Eq. 1) via identical methods to Experiments 1–3. We also analyzed the effect of loudness on illusory tempo via the same methods as before, with the exception that no Huynh–Feldt correction was applied, as there were no violations of sphericity.

**Fig. 7:**
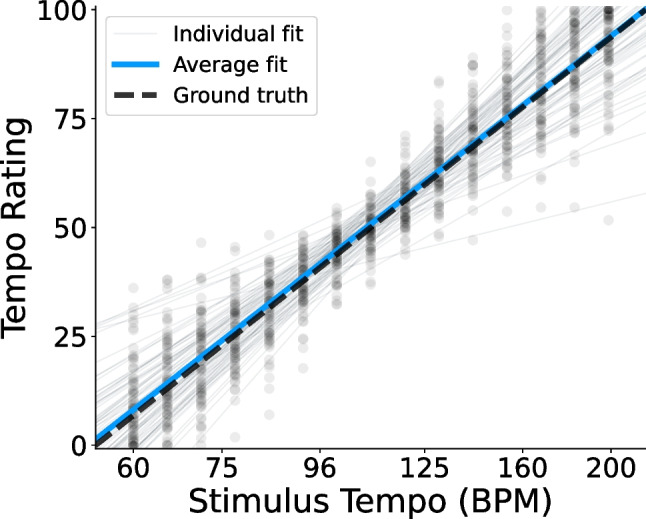
Raw tempo ratings in Experiment 4. *Points* show participants’ average ratings for each stimulus tempo in Experiment 4. Ground-truth ratings (Eq. 1) are indicated by the *dashed line*. Regression lines for individual participants (Eq. 2) appear as thin *gray lines*, and the average of all regression lines appears in *blue*

**Fig. 8:**
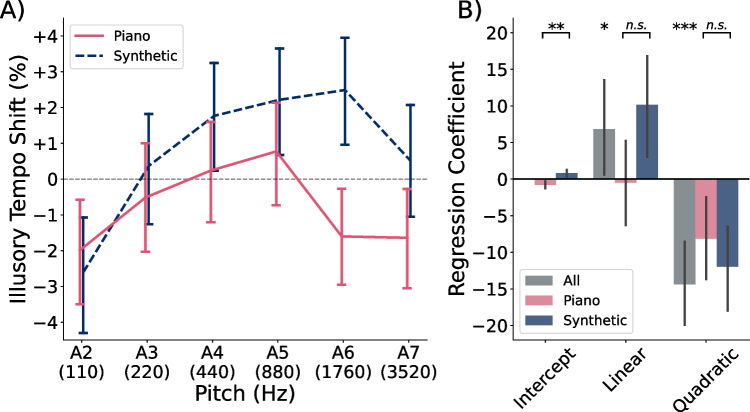
Illusory tempo by pitch and timbre in Experiment 4. **A)** Average illusory tempo shifts across participants for repeating tones of each pitch height and timbre in Experiment 4. Data from piano-tone trials are shown in *pink*, and data from synthetic-tone trials are shown in *blue*. **B)** Average intercepts and slopes of subject-level polynomial regression models, predicting illusory tempo shift as a function of pitch height. *Grey bars* indicate coefficients for models fit to all of a participant’s trials, pooling across timbres. *Pink* and *blue bars* indicate coefficients for models fit only to a participant’s trials of one specific timbre. *Error bars* in both panels indicate 95% confidence intervals. The use of controlled, synthetic tones did not attenuate the quadratic component of the illusory tempo effect

To analyze the main effect of pitch height, we fit subject-level, second-order polynomial regression models to predict illusory tempo shift as a function of pitch height. We then tested the linear and quadratic slopes against zero via a one-sample Hotelling’s $T^2$ test. To analyze the main effect of timbre, as well as whether the effect of pitch differed between timbres, we fit separate second-order polynomial regression models to each participant’s data from each timbre. Then we compared the linear and quadratic slopes, as well as the intercepts, between timbre conditions, using a dependent-samples Hotelling’s $T^2$ test. Significant differences in the intercepts can be understood as a main effect of timbre, whereas significant differences in the slopes can be understood as a significant interaction between pitch height and timbre.

### Results

#### Subjective ratings versus ground truth

Figure 7 illustrates raw tempo ratings as compared to the theoretical ground-truth ratings. The average of all subject-level regression models ($\beta _0=50.74$, $\beta _1=49.41$) did not differ significantly from the ground-truth (Eq. 1), $F(2, 75)=2.13$, $p=.126$, $\eta _p^2=.054$. Participants were highly accurate on average at rating relative tempo.

#### Pitch and timbre

Our primary question of interest in Experiment 4 was whether synthetic tones would eliminate the negative quadratic effect of pitch height on relative tempo judgments. Such a result would suggest that the decrease in tempo ratings at extremely high octaves in Experiments 1–3 may have been due to low-frequency mechanical noise contaminating the piano recordings. Figure 8A shows the average illusory tempo scores for repeating tones of each pitch height, separated by timbre. A clear inverted U-shaped pattern emerged in the perceived tempo of both timbres. The corresponding polynomial regression coefficients are shown in Fig. 8B. Collapsing across timbres, we observed a significant main effect of pitch height on perceived tempo, $F(2, 75)=14.74$, $p<.001$, $\eta _p^2=.282$, which was characterized by both a significant positive linear, $t(76)=2.12$, $p=.037$, $d=0.242$, and negative quadratic slope, $t(76)=-4.87$, $p<.001$, $d=0.555$.

We also observed a significant difference in illusory tempo shift between timbres, $F(3, 76)=5.09$, $p=.003$, $\eta _p^2=.282$; however, this difference was characterized primarily by synthetic tones producing a significantly higher intercept than that of piano tones (i.e., a main effect of timbre), $t(76)=3.38$, $p=.001$, $d=0.720$, and a slightly more positive linear slope, $t(76)=2.20$, $p=.031$, $d=0.361$ (non-significant after correction for multiple comparisons). Rather than eliminate the quadratic effect of pitch height, the synthetic tones showed a more negative average quadratic slope than the piano tones, though not significantly so, $t(76)=-1.00$, $p=.320$, $d=0.153$. These results confirm that the inverted U-shaped effect of pitch height on perceived tempo is not specific to the piano timbre used in Experiments 1–3.

#### Loudness control

We again included a loudness control to assess whether subtle loudness differences between tones may have affected tempo ratings, and found no significant effect of loudness on perceived tempo, $F(2, 152)=0.57$, $p=.567$, $\eta _p^2=.007$. Mean illusory tempo shifts for tones played 3 dB below baseline, at baseline, and 3 dB above baseline were $-0.13\%$ ($SD=2.96$), $+0.36\%$ ($SD=3.20$), and $-0.23\%$ ($SD=2.72$), respectively.

### Discussion

In Experiment 4, we evaluated whether the inverted U-shaped effect of pitch height on tempo judgments was specific to the piano tones used in Experiments 1–3. Specifically, we considered whether the low salience of extremely high pitches (Terhardt et al., 1982) caused participants to focus instead on the low-frequency noise content underlying the highest piano tones. In Experiment 4, we generated synthetic tones without this low-frequency content and compared illusory tempo shifts for tones of both timbres. We found that synthetic tones were perceived as significantly faster than piano tones, but otherwise produced a similar inverted U-shaped effect (see Fig. 8). These results suggest that the non-monotonic shape of the illusory tempo effect is not an artifact of low-frequency content in piano tones, and that the effect may generalize across timbres.

Our results do not rule out the possibility of a direct effect of pitch salience on perceived tempo; they only rule out a confounding effect in which low-frequency sounds from the mechanics of a piano are relatively salient when paired with extremely high-pitched tones. It remains noteworthy that the maximum of our illusory tempo curve falls near the 700-Hz peak of the pitch salience curve of Terhardt et al. (1982). Whether this similarity is a coincidence or evidence for an effect of salience on perceived tempo remains in question. It has been hypothesized that the energy expended in processing a stimulus biases perceived timing (Eagleman, 2008; Eagleman & Pariyadath, 2009), which makes it plausible that pitch salience might influence tempo perception. However, the results of Experiment 5 were inconsistent with this salience account, so we do not address it further.

## Experiment 5

In Experiment 4, the removal of low-frequency noise from high-pitched tones failed to eliminate the negative quadratic effect of pitch on perceived tempo. The origin of this effect thus remains in question. Another possibility is that the quadratic effect is simply a midpoint effect, whereby people perceive the middlemost tones to be the fastest and the most extreme tones to be the slowest. We therefore conducted a fifth experiment in which we divided the pitch range from Experiments 1–4 in half, such that some participants heard only tones between A2 (110 Hz) and D$\sharp$5 (622.3 Hz), and others heard tones only between D$\sharp$5 and A7 (3520 Hz). We subdivided each register into six tones in order to match the six-tone structure of Experiments 1–4. Therefore, if the quadratic effect of pitch represents a midpoint effect, we should observe inverted U-shaped effects in both the lower and upper registers. In contrast, if the quadratic effect is an effect of absolute pitch or pitch salience, we should observe a positive linear effect of pitch in the lower register and a negative linear effect in the upper register.

### Methods

#### Participants

Seventy-six undergraduate students (11 male, 64 female, one non-binary) from McMaster University completed the experiment for course credit. Ages ranged from 17–41 years ($M=19.1$, $SD=3.0$). Participants were randomly assigned to hear a register of lower octaves ($N=37$; 33 female) or a register of upper octaves ($N=39$; 31 female). All participants completed the task online between July and October 2021 due to the COVID-19 pandemic. Three additional participants completed the experiment but were excluded due to low correlation ($r<.5$) between their responses and the true relative tempo (see Response Scoring).

#### Materials

The metronome was identical to that used in Experiments 1–4. Tones were synthetic complex tones constructed in an identical manner to those in Experiment 4. All sound sequences were pre-generated and were played back as WAV files during the experiment.

#### Apparatus

We conducted the experiment online using the same apparatus as Experiments 1–4.

#### Design & procedure

The procedure was identical to that of Experiment 4, with the exception that (1) tones in all six blocks were synthetic and (2) we added a between-subjects manipulation of register. The experiment therefore had a mixed 2 (Register) $\times$ 6 (Pitch Height) $\times$ 15 (Stimulus Tempo) design. Participants assigned to the lower register heard the six pitches A2 (110 Hz), D$\sharp$3 (155.6 Hz), A3 (220 Hz), D$\sharp$4 (311.1 Hz), A4 (440 Hz), and D$\sharp$5 (622.3 Hz), whereas those assigned to the upper register heard the six pitches D$\sharp$5 (622.3 Hz), A5 (880 Hz), D$\sharp$6 (1244.5 Hz), A6 (1760 Hz), D$\sharp$7 (2489.0 Hz), and A7 (3520 Hz).

#### Response scoring

Response scoring was identical to Experiment 4. A total of 664 trials (4.85%) were excluded as outliers.

#### Data analysis

We followed a similar procedure to our prior experiments, in which we first analyzed regression model coefficients together via multivariate tests, then conducted univariate post hoc testing with Holm–Bonferroni correction as necessary. We tested the subject-level linear models of raw tempo ratings, as well as the effect of loudness on illusory tempo shift via the same methods as Experiment 4.

To analyze the effect of pitch height on perceived tempo, we fit subject-level, second-order polynomial regression models to predict illusory tempo shift as a function of pitch height. To assess the main effect of pitch, we tested the linear and quadratic slopes from participants in each register condition against zero via one-sample Hotelling’s $T^2$ tests for each register. To determine whether the effect of pitch differed between registers, we compared the linear and quadratic slopes between registers using an independent-samples Hotelling’s $T^2$ test.

As the average residual tempo and illusory tempo shift are always equal to zero at the subject level, the main effect of register on perceived tempo cannot be assessed by comparing the intercepts of the illusory tempo models of participants in each group. Instead, we compared the intercepts of the raw tempo rating models (Eq. 2) between conditions, using an independent-samples *t*-test. This test captures the main effect of register by determining whether average raw tempo ratings were higher in one register than another.

**Fig. 9:**
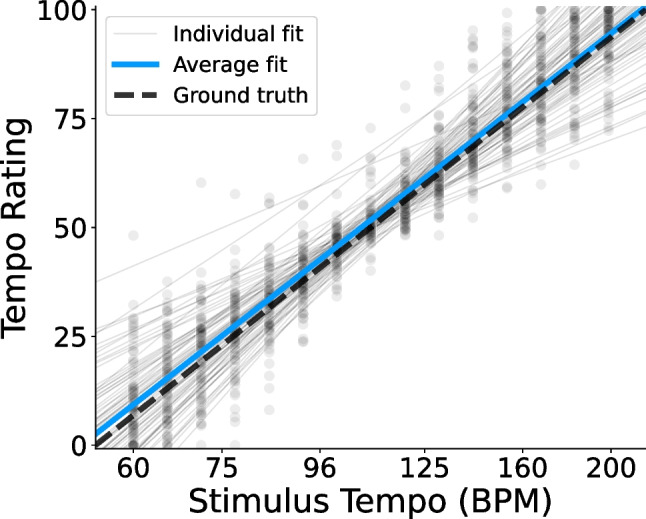
Raw tempo ratings in Experiment 5. *Points* show participants’ average ratings for each stimulus tempo in Experiment 5. Ground-truth ratings (Eq. 1) are indicated by the *dashed line*. Regression lines for individual participants (Eq. 2) appear as thin *gray lines*, and the average of all regression lines appears in *blue*

### Results

#### Subjective ratings versus ground truth

Figure 9 illustrates raw tempo ratings from Experiment 5 as compared with the theoretical ground-truth ratings. The average linear fit ($\beta _0=51.66$, $\beta _1=49.10$) differed significantly from the ground truth, $F(2, 74)=6.91$, $p=.002$, $\eta _p^2=.157$. Similar to Experiments 1–3, the intercept was significantly greater than the ground truth, $t(75)=3.72$, $p<.001$, $d=0.427$, $CI=[50.77, 52.55]$, while the slope did not significantly differ, $t(75)=-0.71$, $p=.479$, $d=0.082$, $CI=[46.57, 51.62]$. Additionally, the average intercept among participants in the upper register ($M=52.16$) was slightly higher than among those in the lower register ($M=51.13$), but not significantly so, $t(74)=-1.15$, $p=.253$, $d=.264$. Participants were generally accurate at judging differences in relative tempo, but tended to slightly overestimate relative tempo overall, regardless of which register they heard.

**Fig. 10:**
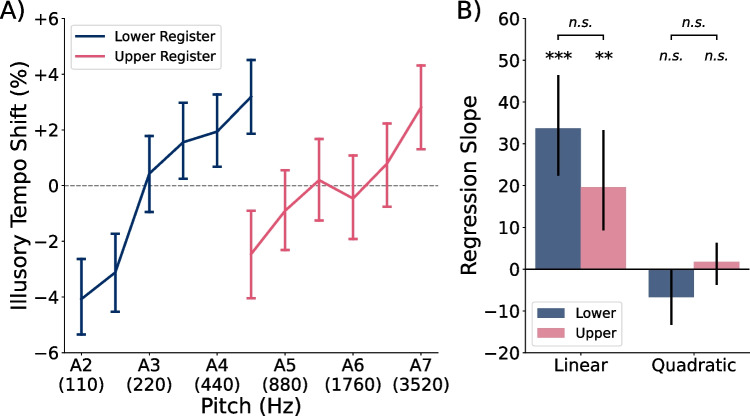
Illusory tempo effects by register in Experiment 5. **A)** *Line plots *indicate illusory tempo shifts across pitch heights in Experiment 5, averaged across participants who heard repeating tones from the lower register only (*blue*) versus the upper register only (*pink*). **B)** Slope coefficients of subject-level polynomial regression models predicting illusory tempo shift as a function of pitch height. *Blue* and *pink bars* show the average coefficients for participants assigned to the lower and upper registers, respectively. *Error bars* in both panels indicate 95% confidence intervals. Participants rated higher pitches as faster in a positive linear pattern, regardless of the register of stimuli they listened to

#### Pitch and register

Figure 10A illustrates illusory tempo shifts from Experiment 5 as a function of pitch height within each register. Contrary to the inverted U-shaped effect observed in Experiments 1–4, the data show a simple higher-equals-faster bias regardless of whether participants heard tones in the lower register only (A2–D$\sharp$5; 110–622.3 Hz) or the upper register only (D$\sharp$5–A7; 622.3–3520 Hz). Multivariate analysis of the regression coefficients (Fig. 10) revealed significant main effects of pitch height in both the lower register, $F(2, 35)=13.41$, $p<.001$, $\eta _p^2=0.434$, and upper register, $F(2, 37)=4.99$, $p=.012$, $\eta _p^2=0.212$. In the lower register, post hoc univariate testing revealed a strong, positive linear effect of pitch on perceived tempo, $t(36)=5.21$, $p<.001$, $d=0.856$, with a nonsignificant quadratic effect, $t(36)=-1.91$, $p=.064$, $d=.315$. In the upper register, we observed a significant positive linear effect of pitch with medium effect size, $t(38)=3.17$, $p=.003$, $d=0.508$, and a nonsignificant quadratic effect of pitch, $t(38)=0.72$, $p=.477$, $d=0.115$. The polynomial regression slopes did not differ significantly between participants assigned to the lower and upper registers (Fig. 10B), $F(2, 73)=2.62$, $p=.080$, $\eta _p^2=.067$, suggesting that pitch height influenced perceived tempo similarly in both registers. In both groups, higher pitches were perceived as faster in a positive linear pattern.

#### Loudness control

We included the same loudness control as in Experiments 1–4 to assess whether subtle loudness differences between tones may have affected tempo ratings, and we again found no significant effect of loudness on perceived tempo, $F(2, 150)=0.45$, $p=.639$, $\eta _p^2=.006$. Mean illusory tempo shifts for tones played 3 dB below baseline, at baseline, and 3 dB above baseline were $+0.19\%$ ($SD=2.07$), $-0.02\%$ ($SD=1.72$), and $-0.17\%$ ($SD=4.04$), respectively.

### Discussion

In Experiment 5, we observed a linear higher-equals-faster bias regardless of whether participants heard tones from lower octaves or upper octaves only. These results are inconsistent with both the hypothesis that the inverted U-shaped effect of pitch depends on a midpoint effect, as well as the hypothesis that it depends on absolute pitch or pitch salience. Rather, our results suggest that the illusory effects of pitch on perceived tempo depend on relative pitch within a context. The finding that register did not significantly affect average raw tempo ratings further supports this importance of relative pitch over absolute pitch.

It also appears that the effect may depend on the range of pitches included in a context. When participants in Experiments 1–4 heard tones spanning a five-octave range, they demonstrated an inverted U-shaped effect of pitch, in which perceived tempo increased with pitch for two to three octaves before reaching a plateau and ultimately declining again. In contrast, when participants in Experiment 5 heard tones spanning only two and a half octaves, perceived tempo simply increased linearly with pitch across the entire range, regardless of whether that context was low or high in absolute pitch. We address potential explanations for this context dependence in the General Discussion, below.

## General discussion

We conducted five experiments to better understand the shape of the relation between pitch and perceived tempo across several octaves. Previous research has identified pitch-induced illusory tempo effects (Boltz, 2011), but only tested these effects within a relatively restricted range of fundamental frequencies (see Fig. 1). Our goal was to map out the shape of these effects across the broader span of human hearing, and to evaluate the generalizability of the claim that higher pitches are perceived as faster. Our results instead suggest a context-dependent effect, in which perceived tempo increases with pitch until peaking between two and three octaves above the lowest pitches in the context. Above this point, the pitch-induced bias reverses direction, resulting in tones five octaves above the lowest pitch being perceived as slower than those two octaves above. In contexts spanning fewer than three octaves, we observed a monotonic higher-equals-faster relationship regardless of the absolute pitch of the context. Furthermore, we found that pitch-induced illusory tempo was consistent regardless of the true tempo, that it was only slightly attenuated via motor synchrony, and that it appeared with both piano tones and synthetic complex tones.

Our results raise the question of why the context’s pitch range matters. One possibility is that the effects we observed are tied to human vocal ranges. Individuals typically have vocal ranges that span about two octaves (Kuhn et al., 1979; Moore, 1991), which is similar to the range over which we observed a bias to rate higher-pitched tones as faster. There are individual and gender differences in the absolute pitch of human voices, so it may be that relative pitch within a speaker or singer’s vocal range better predicts their vocal timing than does the absolute pitch of their voice. Analysis by Broze and Huron (2013) has similarly suggested that relative pitch in Western music better predicts timing than does absolute pitch. Perhaps, then, relative pitch within a context drives illusory tempo because it is informative as a tempo cue. Additionally, as relative pitch may be most meaningful within the range of an individual human voice or typical musical melody, the perceived increase in tempo alongside increasing pitch may primarily apply within a roughly two-octave range.

We are left, then, with the question of how pitch-induced illusory tempo arises, and at which stage of processing. One possibility is that auditory dimensions might interact due to the physics of the peripheral auditory system – similar to interactions between frequency and amplitude that have been observed in the cochlea in the form of frequency detuning (Large, 2010; Ruggero, 1992). Indeed, at least one simulation of auditory nerve and midbrain responses to periodic stimuli has demonstrated a U-shaped effect of stimulus frequency on the strength of neural synchronization to a beat (see Zuk et al., 2018, Supplementary Figure 3). However, the context sensitivity of illusory tempo, as observed in Experiment 5, would be difficult to reconcile with an origin in the dynamics of the auditory periphery.

Therefore, we believe it is most plausible that these pitch-time interactions depend on perceptual experience, as suggested by Boltz (1998), and that they are learned from real-world correlations between pitch and tempo. If these effects do derive from perceptual experience, it may be possible to neutralize or invert the illusion in the short term if, for example, people listened to a selection of music where pitch and tempo were negatively correlated. The developmental trajectory of pitch-time integration also remains an open question, and future research should attempt to measure whether illusory tempo effects are present in infants and young children. Research by Eitan and colleagues on imagined and physical motion while listening to music has at most found weak associations between pitch and movement speed in children as old as 11 (Eitan & Tubul, 2010; Kohn & Eitan, 2009, 2016), suggesting that higher-faster associations may develop later in childhood.

One limitation of our relative tempo judgment task is that it cannot determine whether differences in tempo ratings arise at the level of perception or decision-making. That is, we cannot determine whether participants rated some tones as faster than others because a perceptual bias caused them to hear the tones as faster, or because their decision-making strategy used pitch as a post hoc cue (e.g., “the tones were low, so they were probably slow”). Future work should test perceived tempo as a function of pitch in a task without a decision-making component. One approach would be to use a sensorimotor paradigm, such as the synchronization and continuation tapping tasks used by Boasson and Granot (2012, 2019) and Ammirante et al. (2011), respectively. If pitch-induced illusory tempo arises at the level of perception, participants’ tapping rates should parallel the tempo ratings observed in the present study. However, if these biases arise at the level of decision-making, they should not influence synchrony. Given earlier findings that contour changes, pitch distances, and pitch direction do influence tapping behavior (Ammirante et al., 2011; Boasson & Granot, 2012, 2019), we might expect to find that pitch height also influences the tempo of synchronized motor behavior.

Outside of Experiment 4, we also did not attempt to disambiguate whether our effects derive from the pitch of a sound, versus its frequency content. The perceived pitch of a sound depends not only on its fundamental frequency but also its harmonics, and tones with missing fundamentals and harmonics can still be perceived as having the same pitch (Fletcher, 1924). Future research should test whether perceived tempo varies solely with perceived pitch, or whether identically pitched tones that lack high- or low-frequency harmonics would also be perceived as differing in tempo. Disambiguating the effects of pitch and frequency may help us to identify which stage of perceptual processing gives rise to illusory tempo effects.

Understanding interactions between musical dimensions is an important step towards linking models of pitch and time perception, which have historically been developed independently (e.g., Large et al., 2015; Large et al., 2016), despite evidence for the integration of these processes. We also wish to stress the importance of our findings for research design in auditory perception. Analyses that assume pitch and timing are perceptually independent ignore a growing body of evidence that one can influence the other. It has become standard procedure to control for differences in perceived loudness between pitches (Fletcher & Munson, 1933) when designing experiments. Perhaps pitch-time interactions should be considered, as well.

## Data Availability

All data and materials from our experiments are publicly available on the Open Science Framework at https://osf.io/85cyx/, as well as on GitHub at https://github.com/jpazdera/IllusoryTempo.

## Footnotes

1 To understand this equation, note that when the stimulus tempo is equal to that of the metronome, the relative tempo equals 1, $\log _2(1)=0$, and thus $r_{true}=50$, the intercept of the line. Doubling the stimulus tempo, *t*, doubles the relative tempo, thereby increasing $\log _2(t/t_{ref})$ by 1 and increasing $r_{true}$ by 50, the slope of the line.

2 While a doubling of perceived tempo would correspond to both a $100\%$ increase in BPM and an illusory tempo shift of $+100\%$, a halving of perceived tempo would correspond to a $50\%$ reduction in BPM, but an illusory tempo shift of $-100\%$.

3 We set loudness to be similar to other web-based content, which most streaming platforms normalize to $-14$ LUFS. A difference of $\pm 3$ LUFS is equivalent to the $\pm 3$ dB difference between loudness conditions in Experiments 1–3.

## Code Availability

All code used for running our experiments and processing, analyzing, and visualizing data for the present manuscript is available both on the Open Science Framework at https://osf.io/85cyx/ and on GitHub at https://github.com/jpazdera/IllusoryTempo.
